# Reframing West Nile Virus in Latin America: From Enzootic Evidence to Human Risk—Surveillance Gaps and One Health Actions

**DOI:** 10.3390/v18030281

**Published:** 2026-02-26

**Authors:** Juan S. Izquierdo-Condoy, Janeth C. Gil, Jhan. S. Saavedra-Torres, H. A. Nati-Castillo, Juan Jose Martinez Penaranda, Carolina Vásquez Narváez, Andrés López-Cortés, Marlon Arias-Intriago, Esteban Ortiz-Prado

**Affiliations:** 1 One Health Research Group, Universidad de Las Américas, Quito 170124, Ecuador; 2 Facultad de Salud, Universidad Santiago de Cali, Cali 760033, Colombia; 3 Grupo de Investigación en Salud (GIS), Departamento de Medicina Interna, Universidad del Cauca, Popayán 190001, Colombia; 4 Grupo de Investigación en Biomateriales y Biotecnología—BEO, Facultad de Salud, Universidad Santiago de Cali, Cali 760033, Colombia; 5 Interinstitutional Internal Medicine Group (GIMI 1), Universidad Libre, Cali 760032, Colombia; 6 Facultad de Salud, Pontificia Universidad Javeriana, Cali 760031, Colombia; 7 Cancer Research Group (CRG), Universidad de Las Américas, Quito 170124, Ecuador; 8 Pathology Department, SOLCA Quito, Quito 170518, Ecuador

**Keywords:** West Nile virus, arboviruses, One Health, surveillance systems, climate change, Latin America

## Abstract

West Nile virus (WNV) is a mosquito-borne flavivirus with one of the widest global distributions. Since its discovery in Uganda in 1937, it has become a major zoonotic pathogen, and after its introduction into the United States in 1999, it spread rapidly across the Americas, becoming the leading cause of neuroinvasive arboviral disease. Its expansion illustrates a remarkable ecological adaptability, further intensified by climate change. In Latin America and the Caribbean, WNV circulation has been consistently documented in birds, horses, and mosquitoes; however, confirmed human cases remain disproportionately scarce compared with North America and Europe. Reports include sporadic human cases in Brazil (>100 since 2014), Mexico (~13), Argentina (2006–2007), Puerto Rico (2007), Nicaragua, and Haiti, while animal and vector evidence extends to Guatemala, El Salvador, Belize, Costa Rica, Bolivia, Paraguay, Colombia, Venezuela, Cuba, and Ecuador. This paradox likely reflects structural limitations within regional health systems, including underdiagnosis, restricted diagnostic capacity, and significant surveillance gaps, particularly in contexts where mild febrile syndromes may be misclassified as dengue, Zika, or Chikungunya. The regional risk of emergence is further amplified by climatic variability, ecological change, and intensifying human–wildlife interactions. Experiences from Europe highlight the importance of early detection, transfusion safety, and integrated surveillance within a One Health framework. Strengthening preparedness in Latin America will require investments in diagnostic infrastructure, implementation of standardized seroepidemiological surveys, development of predictive models tailored to local ecological contexts, and robust intersectoral collaboration.

## 1. Introduction

West Nile virus (WNV) is a mosquito-borne, zoonotic flavivirus and one of the most widely distributed arboviruses globally. It was first isolated in 1937 from the blood of a febrile patient in the West Nile District of Uganda and belongs to the Japanese encephalitis virus serocomplex, which also includes St. Louis encephalitis virus (SLEV), Murray Valley encephalitis virus (MVEV), and Alfuy virus (ALFV) [1,2].

Because West Nile virus and St. Louis encephalitis virus belong to the same Japanese encephalitis serocomplex, serological cross-reactivity driven by conserved epitopes is common and represents a major diagnostic challenge in flavivirus-endemic regions [3,4]. Clinical presentation alone is insufficient to reliably distinguish between these infections, in this context, initial screening is often based on IgM or IgG enzyme-linked immunosorbent assays; however, definitive differentiation between WNV and SLEV requires confirmatory plaque reduction neutralization tests (PRNTs), which remain the reference standard [5]. Molecular methods, including RT-PCR and genomic sequencing, allow specific identification during the acute viremic phase but are limited by the short duration of detectable viremia [6].

Initially regarded as a pathogen of limited human relevance due to its predominantly mild or subclinical presentations, WNV is now recognized as a major public health threat capable of causing significant morbidity and mortality in humans and a broad range of vertebrates, including birds, horses, reptiles, and small mammals [7].

The global epidemiology of WNV has shifted markedly in the past decades. In the Americas, following its introduction into the United States in 1999, the virus spread rapidly across the continent, becoming the leading cause of neuroinvasive arboviral disease [8]. Between 1999 and 2017, nearly 22,999 neuroinvasive cases were reported in the U.S., arising from millions of infections [9]. In Europe, the Middle East, and Russia, multiple large outbreaks have occurred, including the unprecedented 2018 European epidemic, which resulted in more than 1900 human cases [10]. More recently, autochthonous infections have been documented in regions previously unaffected, such as Germany and the Netherlands, underscoring the expanding ecological niche of WNV [11].

Recent epidemiological transitions highlight the remarkable adaptability of WNV to changing environments [12]. In Greece, evidence of overwintering in active mosquito populations during 2022 raised the possibility of year-round circulation in temperate regions [13]. Similarly, unusually high numbers of neuroinvasive cases reported in Israel as early as June 2024 illustrate how warmer winters and springs may accelerate viral amplification [14]. Modeling studies in southern Europe have projected that rising temperatures extend the seasonal window of transmission, while in the U.S., precipitation patterns and large-scale spatial synchrony of mosquito populations have been linked to outbreak intensity [15]. Collectively, these findings position climate change as a critical driver of WNV dynamics, reshaping vector distribution and geographic spread.

In contrast, in Latin America and the Caribbean, WNV circulation has been repeatedly documented in vectors and animal hosts, yet the number of confirmed human cases remains disproportionately low compared with North America and Europe. This paradox refers to the marked contrast between the extensive and sustained enzootic circulation of West Nile virus documented in birds, horses, and mosquito vectors across Latin America and the Caribbean, and the disproportionately low number of confirmed human cases reported in the region, particularly when compared with North America and Europe. This discrepancy raises critical questions regarding underdiagnosis, ecological and immunological factors, and structural gaps in regional surveillance capacity. Against this backdrop, this article aims to provide a comprehensive overview of current evidence on WNV infection in Latin America, and to examine its broader implications for public health and One Health strategies in the region.

## 2. Virology and Transmission Dynamics

The WNV is maintained in nature through a bird–mosquito transmission cycle. Birds act as the principal amplifying hosts, sustaining high-titer viremia that enables efficient transmission to mosquitoes, particularly those of the genus *Culex* [16]. In contrast, humans and horses are considered dead-end hosts, as their viremia levels are insufficient to perpetuate the cycle [17]. While mosquito bites remain the predominant route of transmission, alternative pathways have been documented in experimental and clinical contexts, including blood transfusion, organ transplantation, intrauterine transmission, and breastfeeding [1]. Viral shedding in urine further suggests that environmentally mediated transmission may be possible.

In the Americas, *Cx. pipiens*, *Cx. quinquefasciatus*, and *Cx. tarsalis* are regarded as the most competent vectors in North America, where their abundance in urban and peri-urban environments coincides with epidemic peaks [18]. In Latin America, *Cx. quinquefasciatus* and related species are widely distributed and ecologically suited to sustain WNV transmission [16]. Studies in Mexico and Central America suggest that viremic migratory birds, together with the widespread presence of *Culex* mosquitoes, facilitate regional introduction and persistence of the virus [7]. The detection of *Culex* mosquitoes in international airports further highlights the potential for human-mediated dispersal [19].

From a genetic perspective, WNV comprises multiple lineages, but only lineages 1 and 2 are considered of major global relevance [20]. Lineage 1 is the most widespread, associated with outbreaks across Africa, Europe, the Middle East, and the Americas. Lineage 2, once restricted to sub-Saharan Africa, has emerged as an important cause of human disease in Europe [1,21]. Experimental and field evidence suggests that lineage-specific differences in virulence, vector competence, and ecological adaptation may partially explain the observed geographic heterogeneity in human disease incidence.

## 3. Current Evidence in Latin America and the Caribbean

### 3.1. Circulation in Animals and Vectors

Since the early 2000s, circulation of WNV in Latin America and the Caribbean has been primarily documented through findings in animals and vectors. The first confirmed isolation in South America occurred in Argentina in 2006 from horses with encephalitis, where viral isolation and molecular detection provided definitive evidence of active infection, marking the official introduction of the virus into the region [22]. Since then, multiple serological studies, most commonly based on the detection of neutralizing antibodies, have identified evidence of WNV exposure in resident birds, horses, non-human primates, and reptiles, often with confirmation by plaque reduction neutralization tests (PRNTs) to address flavivirus cross-reactivity, as well as viral isolations from mosquitoes, confirming active transmission in diverse ecosystems [23].

In Mexico, the evidence is broad and consistent, serological surveys have detected antibodies against WNV have been identified in wild birds, reptiles, and mammals, and the virus has been isolated from *Culex quinquefasciatus* and other species, suggesting established enzootic circulation [24,25]. In the Caribbean, monitoring studies conducted after the first introductions from North America confirmed local transmission: resident birds from Puerto Rico and Cuba showed neutralizing antibodies to WNV confirmed by PRNT, ruling out infections solely in migratory species [26].

In Central America, evidence is also strong. In Guatemala (2005–2008), WNV was isolated from *Culex quinquefasciatus* and annual circulation was detected through entomological studies, although without clinical evidence in humans [27]. In El Salvador, between 2001 and 2003, an epizootic caused the death of 203 horses; subsequent analyses demonstrated that 25% of surviving stablemates had antibodies against WNV, with 10 confirmed by PRNT, thereby confirming the expansion of the virus into Central America [28]. In Belize, an early 2003 report documented a clinically infected horse with laboratory confirmation, constituting the first evidence of circulation in the country [29]. In Costa Rica, serological studies using WNV-specific assays, with confirmatory neutralization testing, have shown co-circulation of flaviviruses in birds, horses, and wild primates, with specific detection of neutralizing antibodies against WNV in some animals, though no human cases have been reported to date [30,31].

**Figure 1:**
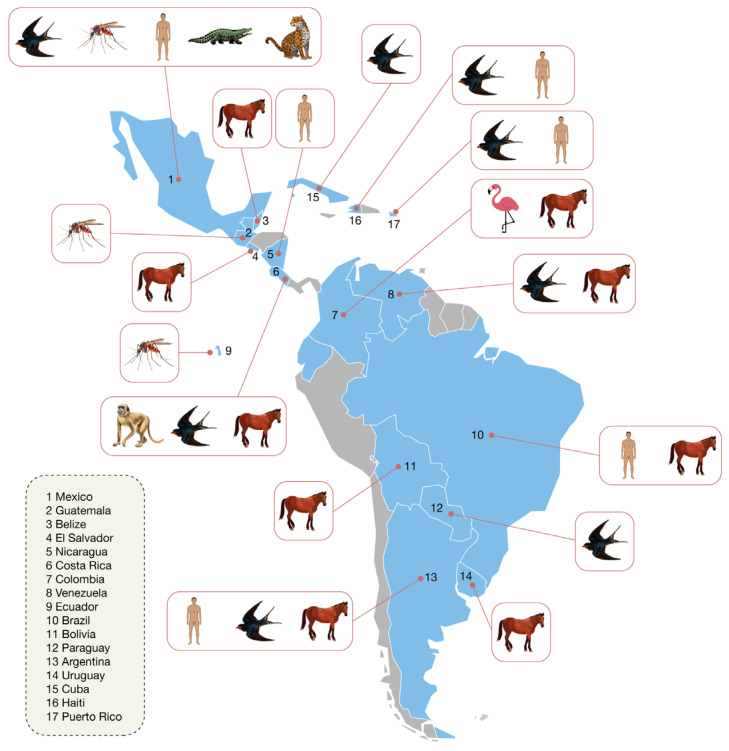
Summary of WNV evidence distribution in Latin America and the Caribbean (2001–2024). This figure summarizes evidence of WNV across Latin America and the Caribbean from 2001 to 2024. Icons denote the type of finding by country (human, horse, bird, mosquito, reptile, mammal). For vectors, the “mosquito” icon refers primarily to *Culex quinquefasciatus*, with virus isolations in Guatemala (2005–2008) and demonstrated vector competence for Galápagos (Ecuador) strains. In Mexico, beyond isolation in *Cx. quinquefasciatus*, neutralizing antibodies have been documented in resident birds, reptiles (*Crocodylus acutus* and *C. acutus* × *C. moreletii* hybrids), and mammals (e.g., *Panthera onca* or jaguar), *Canis latrans* or coyote), indicating heterogeneous enzootic circulation. Among equines, evidence includes the El Salvador epizootic (2001–2003), a single horse case in Belize (2003), seropositivity in Bolivia, and in Argentina both seropositivity and virus isolation in 2006; Colombia and Venezuela also report equine seropositivity. In birds, WNV has been confirmed in Paraguay (*Thamnophilus doliatus*), with neutralizing antibodies in Cuba (resident birds) and seropositivity reported in Colombia and Venezuela. Regarding humans, confirmed cases have been reported in Brazil (>100; 2014–2024), Mexico (~13), Argentina (2006–2007), Puerto Rico (2007), Nicaragua (anti-prM serologic evidence in children with infections predating 2007–2009), and Haiti (two historical cases). The figure synthesizes evidence published in peer-reviewed articles and technical reports for the indicated period.

In South America, beyond Argentina, circulation has been documented in several countries. In Bolivia, a 2011 study showed that 59.4% of 160 horses had antibodies using competitive ELISA, one-third of which were confirmed by PRNT, with no evidence of recent infections [32]. In Paraguay (2016–2018), serological surveys in resident birds identified neutralizing antibodies against WNV in a barred antshrike (*Thamnophilus doliatus*), representing the first serological evidence of the virus in the country [33]. In Colombia and Venezuela, multiple studies have demonstrated circulation in birds, horses, and other animals, including antibodies in captive flamingos and equines, largely through serological detection confirmed by neutralization assays, although no confirmed human cases have been reported [34,35,36]. In Ecuador, *Culex quinquefasciatus* populations from the Galápagos Islands have been shown experimentally competent to transmit WNV, and serological studies in horses have so far yielded negative results, reflecting potential but not yet established circulation [16] (Figure 1).

### 3.2. Human Cases: Confirmed but Limited

Unlike North America and Europe, Latin America has not recorded large-scale human epidemics of WNV. Confirmed cases remain sporadic, totaling only a few dozen across the region [23,24].

In Brazil, a recent multicenter study reported 110 confirmed cases between 2014 and 2024 in 13 states, with clinical presentations including febrile and neuroinvasive forms, and four fatalities [37]. Cases were identified using serological assays (IgM/IgG) complemented by molecular detection (RT-PCR) in acute presentations, and the first internationally confirmed case occurred in 2015 in Piauí, associated with acute flaccid paralysis [38]. In Mexico, despite extensive circulation in animals, only 13 human cases were confirmed between 2002 and 2023, confirmation relying on RT-PCR or viral isolation in early cases and serology with neutralization testing in later investigations, with the first viral isolation in 2005 and a fatal case in 2009 [24,25,39,40].

In Argentina, sporadic human cases were reported in 2006–2007 across at least five northeastern and central provinces, diagnosed primarily through serological assays and, in selected cases, molecular confirmation, coinciding with the first equine isolation of the virus in South America [41]. In Nicaragua, a pediatric cohort study identified anti-prM antibodies to WNV in three children, with infections predating 2007–2009, providing the first human evidence of WNV in Central America [42].

In the Caribbean, Puerto Rico documented human transmission in 2007 through laboratory detection in a blood donor and a symptomatic patient, using serological screening with confirmatory testing, within a context of active surveillance that also included mosquitoes and raptors [43]. In Haiti, a population-based study detected IgG antibodies against WNV by serological testing in 7 of 673 participants (≈1%), although cross-reactivity with dengue was suggested; historically, only two human WNV cases have been confirmed in the country [44] (Figure 1).

Available data suggest that the clinical spectrum of human WNV infection in Latin America mirrors that observed in other regions, ranging from asymptomatic infection and mild febrile illness to neuroinvasive disease, including meningitis, encephalitis, and acute flaccid paralysis [45]. However, systematic characterization of disease severity in the region remains limited. In Brazil, where the largest number of confirmed cases has been reported, both febrile and neuroinvasive presentations have been documented, with a small number of fatalities. In other countries, human cases have been identified primarily through targeted investigations, blood donor screening, or retrospective serological studies, rather than through routine clinical surveillance [46,47,48]. The apparent scarcity of reported neuroinvasive disease likely could reflect a scenario of limited case detection and reporting, rather than true absence of severe infection in Latin America, underscoring the need for improved clinical awareness and surveillance integration in the region.

## 4. Public Health Implications

The panorama of WNV in Latin America is paradoxical: while serological and entomological evidence confirms widespread circulation in wildlife and vectors, confirmed human cases remain rare [23]. This discrepancy likely reflects substantial underreporting and diagnostic limitations, as mild febrile cases may be misclassified as dengue, Zika, or Chikungunya [49,50]. Although notification of WNV is officially mandatory across nearly all countries in the region (Table 1), human surveillance is predominantly passive and restricted to neurological or encephalitic syndromes, which hampers timely detection of sporadic or mild cases.

In addition to underdiagnosis and surveillance limitations, several complementary hypotheses may also contribute to the relatively low number of reported human West Nile virus cases in Latin America. These include partial cross-protective immunity resulting from high background exposure to other endemic flaviviruses, such as dengue, Zika, and St. Louis encephalitis virus, which may modulate susceptibility to symptomatic or neuroinvasive WNV infection [69]. Differences in vector competence, feeding behavior, and host preference among Culex species circulating in the region may further influence the likelihood of human exposure and transmission [70]. Moreover, lineage-specific viral characteristics and host–virus interactions can shape pathogenicity and neuroinvasive potential, contributing to heterogeneity in clinical presentation and detection [71,72]. Importantly, these mechanisms are not mutually exclusive and likely interact with structural diagnostic and surveillance gaps, collectively shaping the observed epidemiological pattern.

From a public health perspective, the consequences are significant. Climate change acts as a critical driver by expanding the habitats of *Culex* mosquitoes, shortening the extrinsic incubation period of the virus, and altering bird migration patterns [73]. Experiences in Europe illustrate these risks: in the Netherlands and Germany, climatic and land-use changes have been linked to outbreaks of WNV and Usutu virus (USUV) [74,75]. Such findings suggest that viral expansion into new areas of Latin America is both possible and likely underestimated.

Another concern is transfusion-related transmission, already documented in North America and Europe, which led to the implementation of nucleic acid testing (NAT) in blood donations in affected regions [76,77]. Similar measures may become essential in Latin America, particularly if viral activity in animals or vectors is detected near blood collection centers.

Finally, European experience highlights that integrated surveillance under a One Health framework—combining entomological monitoring, veterinary surveillance, and human case detections—is the most effective approach to anticipate outbreaks. Italy’s regional programs, initiated in 2008 and later scaled nationally, successfully correlated mosquito, bird, and human data to guide real-time control measures. Comparable models in the Netherlands, involving citizen science, zoos, and migratory bird sampling, proved crucial for early detection and prediction [78].

In Latin America, however, integrated approaches remain incipient. The documented circulation in fauna, favorable environmental conditions, and fragmented surveillance systems suggest that human outbreak risk is underestimated. Strengthening intersectoral collaboration across veterinary, public health, and conservation sectors is fundamental to ensure early detection of new transmission areas, safeguard transfusion safety, and reduce the probability of large-scale epidemics in the region [49].

## 5. Research and Surveillance Priorities in a One Health Framework

Building on the implications above, we propose a shift from fragmented activities to an integrated One Health preparedness agenda that couples technical rigor with community engagement and intersectoral governance.

### 5.1. Population-Based Seroepidemiological Studies

Implement standardized, population-based serosurveys—stratified by eco-regions and urban/rural gradients—to estimate true infection prevalence, including asymptomatic and mild infections frequently misclassified as other arboviruses [79]. Incorporate community-based sampling and participatory designs to expand coverage in hard-to-reach settings and reduce selection bias.

### 5.2. Strengthening Diagnostic Capacity and Clinical Awareness

Among the proposed research and surveillance priorities, strengthening diagnostic capacity across public health and animal health sectors represents the highest-yield and most immediately actionable intervention in resource-limited settings. Improved diagnostic availability underpins early case detection, accurate burden estimation, timely outbreak response, and the effectiveness of all subsequent surveillance, modeling, and preparedness efforts [80,81]. Therefore, investments in diagnostic capacity should precede or accompany all other One Health interventions aimed at mitigating the risk of WNV emergence. Overcoming current molecular and serological capacity gaps, as well as the cross-reactivity that complicates case attribution in flavivirus-endemic areas, is essential. Key priorities include expanding access to RT-PCR and genomic sequencing, validating highly specific serological platforms (e.g., PRNT), and investing in decentralized hub laboratories linked to national reference centers to accelerate confirmation and reporting [12].

Concurrently, clinical awareness should be strengthened to raise suspicion for WNV in both febrile and neurological syndromes and to improve differentiation from dengue, Zika, and chikungunya. This requires targeted training of interdisciplinary teams—including clinicians, neurologists, microbiologists, veterinarians, entomologists, and public health officers—alongside community leaders, focusing on case recognition, biosafety, sampling, and timely notification [1].

### 5.3. Predictive Climate and Ecological Modeling

Develop region-specific predictive models that integrate temperature, precipitation, land-use change, *Culex* dynamics, and bird migration/phenology to anticipate hotspots and guide seasonal vector control, transfusion safety policies, and resource allocation. Lessons from the Mediterranean Basin show that climate anomalies (e.g., warmer springs, earlier seasonal peaks) can precede major outbreaks. Incorporate local and ancestral knowledge (e.g., phenological cues, shifts in breeding sites) as qualitative covariates to improve model performance and operational relevance [82].

### 5.4. Toward Integrated One Health and Community Surveillance

As demonstrated in Europe and the Mediterranean, surveillance is most effective when human, animal, and vector data are integrated. In Latin America, establish multisectoral and transnational frameworks to enable early viral detection, enhance transfusion safety, and generate actionable indicators for decision-makers. Define a One Health governance architecture (roles, decision flows, accountability) with sustainable financing (contingency funds and climate-linked lines) and deploy regional dashboards with near-real-time data and cross-border sharing agreements [7,75].

In parallel, co-design surveillance with community organizations and Indigenous peoples (“salud propia”), leveraging citizen science to report sick/dead birds, map Culex breeding sites, and document environmental signals, and provide multilingual materials—including Spanish and Portuguese, as well as relevant Indigenous languages in settings where Indigenous and rural communities play a central role in environmental monitoring and early detection—to increase timely reporting, acceptability, and local ownership of surveillance activities [41].

## 6. Conclusions

WNV has undergone a marked transition, evolving from a pathogen once regarded as of limited relevance to humans into a global zoonotic threat, characterized by a wide geographic distribution and a remarkable capacity to adapt to changing ecological and climatic conditions. While its epidemiological impact has been clearly documented in regions such as North America and Europe, the picture in Latin America remains less well defined. Here, consistent evidence of viral circulation in vectors and animal reservoirs has been reported across several countries, yet confirmed human cases remain unusually scarce. This discrepancy likely reflects regional health system limitations, including underdiagnosis, constrained diagnostic capacity, and significant gaps in surveillance.

The convergence of climate change, ecological modifications, and increasing human–wildlife interactions amplifies the risk of WNV emergence and expansion into new territories of Latin America and the Caribbean. Lessons from Europe underscore the importance of early detection, transfusion safety, and the integration of entomological, veterinary, and human data into surveillance programs. Strengthening regional preparedness through a One Health framework is essential to confront WNV, requiring investments in diagnostic infrastructure, the implementation of standardized seroepidemiological surveys, and the development of predictive models tailored to local ecological contexts.

## Figures and Tables

**Table 1:** Surveillance systems of WNV in Latin America countries.

Country	Mandatory Notification	Human Surveillance	Animal/Vector Surveillance
Argentina [51]	Yes (official, SENASA/MoH)	Passive; 1 autochthonous case (2023)	Focal active (horses/birds)
Bolivia [52]	Yes (emerging zoonosis)	Passive (neurological syndrome)	Limited active (entomology/animals)
Brazil [53]	Yes (compulsory, immediate, 2017)	Passive (SINAN, neurological syndromes)	Integrated active (birds, horses, mosquitoes)
Chile [54]	Yes (since 2005)	Passive (encephalitis/meningitis)	Focal active (travelers/migratory birds)
Colombia [55]	Yes (exotic event, OIE/INS)	Passive (neuroinvasive, SIVIGILA)	Partial active (horses, vectors, ICA)
Costa Rica [56]	Yes (mandatory reporting)	Passive (encephalitis/meningoencephalitis)	Limited active (horses/birds)
Cuba [57]	Yes (national system)	Passive (suspected cases)	Active (preventive entomology)
Dominican Republic [58]	Yes (mandatory event, regional)	Passive (neurological cases)	Targeted active (animals/vectors)
Ecuador [59]	Yes (SIVE-Alert)	Passive (encephalitis suspicion)	Limited active (horses/wetlands)
El Salvador [60]	Yes (since regional emergence)	Passive (viral encephalitis)	Focal active (mosquitoes)
Guatemala [61]	Yes (mandatory reporting)	Passive (neurological cases)	Targeted active (horses/cattle)
Honduras [62]	Yes (arboviruses)	Passive (viral encephalitis)	Active (entomology, regional control)
Mexico [63]	Yes (NOM-017 and NOM-032, 2003)	Passive (suspected cases, SINAVE)	Active (birds, horses, wildlife)
Nicaragua [64]	Yes (humans and animals, 2016)	Passive (encephalitis/meningitis)	Active (horses, birds)
Panama [65]	Yes (emerging diseases)	Passive (viral encephalitis)	Active (birds, sentinel horses)
Paraguay [66]	Yes (Resolution S.G. 190/2013)	Passive (neurological viral syndrome)	Limited active (birds, horses)
Peru [67]	Yes (SENASA, since 2008)	Passive (encephalitis; no local cases)	Active (horses, birds, testing)
Uruguay [68]	Yes (Group A, immediate)	Passive (<24 h suspected cases)	Limited active (horses, birds)

This table summarizes the official status of WNV as a notifiable disease across Latin American countries, as well as the predominant modalities of surveillance in humans and animals/vectors. While notification is legally mandated in nearly all countries, human surveillance is predominantly passive and focused on neurological or encephalitic syndromes, with only isolated confirmed cases reported (e.g., Argentina 2023, Brazil 2014–2024). In contrast, animal and vector surveillance is variable, ranging from limited or focal activities (e.g., targeted entomology or sentinel equine/bird sampling) to more integrated approaches (e.g., Brazil, Mexico). The heterogeneity of surveillance systems highlights important gaps that may contribute to underdiagnosis and underreporting of WNV in the region.

## Data Availability

This study did not generate new datasets. All information analyzed is available in the published literature referenced in this article.

## Abbreviations

The following abbreviations are used in this manuscript:

**Abbreviation**	**Definition**
ALFV	Alfuy virus
CC BY	Creative Commons Attribution
CDC	Centers for Disease Control and Prevention
CRG	Cancer Research Group
Cx.	*Culex*
ELISA	Enzyme-Linked Immunosorbent Assay
GIS	Grupo de Investigación en Salud
GIMI	Interinstitutional Internal Medicine Group
ICA	Instituto Colombiano Agropecuario
IgG	Immunoglobulin G
INS	Instituto Nacional de Salud
MoH	Ministry of Health
MVEV	Murray Valley encephalitis virus
NAT	Nucleic Acid Testing
OIE	World Organisation for Animal Health
PAHO	Pan American Health Organization
PRNT	Plaque Reduction Neutralization Test
RT-PCR	Reverse Transcription Polymerase Chain Reaction
SENASA	Servicio Nacional de Sanidad y Calidad Agroalimentaria
SINAN	Sistema de Informação de Agravos de Notificação
SINAVE	Sistema Nacional de Vigilancia Epidemiológica
SIVIGILA	Sistema Nacional de Vigilancia en Salud Pública
SLEV	St. Louis encephalitis virus
USUV	Usutu virus
WHO	World Health Organization
WNV	West Nile virus
